# Effects of a moderate combined exercise program on cognitive function, quality of life, and glycemic profile in older adults with type II diabetes mellitus

**DOI:** 10.1016/j.clinsp.2025.100833

**Published:** 2025-11-19

**Authors:** Matheus Henrique dos Santos Lino, Alisson de Lima Guiotto, Vanderlei Carneiro da Silva, Marcus Vinicius Grecco, André Luiz de Seixas Soares, Catherine L. Davis, José Maria Soares-Junior, Edmund Chada Baracat, Guilherme Carlos Brech, Adriana Machado Saldiba de Lima, Júlia Maria D’Andréa Greve, Angelica Castilho Alonso

**Affiliations:** a Graduate Program in Aging Sciences, Universidade São Judas Tadeu (USJT), São Paulo, SP, Brazil; b Researcher at the Movement Study Laboratory, Hospital das Clínicas da Faculdade de Medicina da Universidade de São Paulo (FMUSP), São Paulo, SP, Brazil; c Georgia Prevention Institute, Medical College of Georgia, Augusta University, Augusta, GA, USA; d Department of Obstetrics and Gynecology, Gynecology Discipline, Hospital das Clínicas da Faculdade de Medicina da Universidade de São Paulo (FMUSP), São Paulo, SP, Brazil

**Keywords:** Longevity, Quality of life, Cognition, Diabetes mellitus, type 2, Exercise

## Abstract

**Background:**

The risk of developing non-communicable chronic diseases, such as Type 2 Diabetes Mellitus (T2DM), has increased due to factors such as sedentary behavior, the adoption of nutrient-poor dietary patterns, and an increase in population longevity. Regular physical activity has been shown to lower these risks and prevent chronic non-communicable diseases.

**Objective:**

This study aims to assess how a combined training program affects the Quality of Life (QoL), cognitive function, and glycemic profile of older people with T2DM.

**Methods:**

Forty-three senior citizens participated in this experimental study. The process of assessing the glucose profile involved drawing 20 milliliters of peripheral blood for laboratory examination. The WHOQOL-Old and WHOQOL-Bref questionnaires were used to assess QoL, and the Montreal Cognitive Assessment (MoCA) was used to measure cognitive function. Targeting the main muscular groups, the intervention includes combination training for 12-weeks, twice a week, comprising leg press, calf raises, chest press, rowing, leg curls, and abdominal exercises. There was also thirty minutes of moderate intensity aerobic cycling exercise.

**Results:**

After three months of moderate CT, older adults with T2DM showed improvements in fructosamine levels (partial η² = 0.07), indicating a medium effect size. Small effect sizes (partial η² ≈ 0.02–0.04) were observed in QoL domains related to physical, psychological, and environmental well-being. In cognitive function, a significant improvement was found in the abstraction domain (p = 0.031; partial η² = 0.12), with additional small effect sizes in delayed recall (partial η² = 0.04) and orientation (partial η² = 0.04) domains.

**Conclusion:**

There was a reduction in fructosamine levels and slightly improved QoL in physical, psychological, and environmental domains, with no change in HbA1c or aging-specific QoL. Cognitive function showed improvement in abstraction and small gains in recall and orientation.

## Highlights

• Type 2 Diabetes Mellitus and quality of life.
• Combined training as a treatment for cognitive decline.
• Physical exercise as a therapeutic approach for people with Type 2 Diabetes Mellitus.

## Introduction

The risk of developing non-communicable chronic diseases, such as Type 2 Diabetes Mellitus (T2DM), has increased dramatically due to factors such as sedentary behavior, adoption of nutrient-poor dietary patterns, and an increase in population longevity. This ailment has become dangerously more prevalent, raising major public health concerns. T2DM may impact the Quality of Life (QoL) and cognitive function as people age, especially in older adults.^1^^,^^2^

People living with T2DM have a lower QoL than those without this condition. Diabetes-related disorders, including heart disease, renal problems, neuropathy, and visual problems, have a detrimental effect on QoL. People with T2DM also have to follow diets to check their blood sugar levels and modify their lifestyles, which can be difficult, particularly if unmanaged. Concerns about the future, such as dread of long-term problems, might arise from polypharmacy, especially when it has unwanted side effects, and the diagnosis of a chronic illness.^3^, ^4^, ^5^

Researchers and healthcare practitioners have acknowledged the difficulties of T2DM problems affecting the Central Nervous System (CNS) for more than a century. The frequent complaints from people with T2DM that point to a decline in memory and attention are indicative of this. The currently available research substantially associates T2DM with both brain structural changes and decreased performance in a variety of cognitive domains.^6^

In this context, studies have shown that physical activity and/or exercise can lower risks and prevent diseases like cardiovascular and cerebrovascular diseases, hypertension, T2DM, osteoporosis, obesity, anxiety, and depression. Additionally, it lessens the chance of falls and fall-related injuries. Additionally, Gremeaux et al. (2012)^7^ show that physical activity improves sleep quality and can either prevent or postpone cognitive deterioration.

Combined Training (CT) involves performing both aerobic and strength exercises in the same training session, leading to significant improvements in biochemical variables, glycemic control, and strength. Regular physical exercise has several benefits for mental and cognitive health, particularly in reducing age-related neurodegenerative declines, according to the systematic review accompanied by Jia et al. (2019).^8^

Due to its balanced effectiveness and safety, moderate-intensity CT is more beneficial for older adults with T2DM than light or intense training. Without the significant risks of musculoskeletal injuries and cardiovascular events associated with intense exercise, moderate exercise significantly improves insulin sensitivity, glycemic control, and cardiovascular fitness. Furthermore, moderate intensity can be maintained over time, promoting continued engagement in the exercise program. Moderate exercise intensity offers significant improvements in metabolic and cognitive health when compared to light exercise, which makes it optimal for managing T2DM.^9^

While the effects of exercise on T2DM management are well-documented,^10^ comprehensive studies on the comparative effectiveness of CT remain lacking. More specifically, moderating factors like exercise frequency and intensity are not well-analyzed in terms of their unique effects on the QoL and cognitive function of older adults with T2DM. Zhang et al.'s systematic review from 20,23^9^ shows that CT significantly increased the physical domain of QoL, albeit with a high degree of heterogeneity (*I*^2^ = 84 %). In the psychological domain, where there was no improvement and high heterogeneity (*I*^2^ = 90 %), sensitivity analysis was not carried out because of the small number of studies. Exercises were beneficial to enhancing cognition in the T2DM population; however, because of the heterogeneity of the instruments and the small number of studies, a meta-analysis could not be carried out.

Despite the benefits of exercise in managing T2DM having been documented, there are still uncertainties regarding how effective CT is in comparison, particularly concerning how it affects the QoL and cognitive function of older people with this condition. Comprehensive studies on this subject are lacking, which emphasizes the importance of additional studies to fill the knowledge gap and offer reliable evidence of the advantages of CT in T2DM patients.^9^

Therefore, the current study was designed to assess how a CT program affects the glycemic profile, cognitive function, and QoL of older people living with T2DM.

## Methods

### Study design and setting

This is an experimental study conducted in São Judas Tadeu University (USJT) in collaboration with the Movement Study Laboratory (LEM) at the Hospital das Clínicas of São Paulo's Institute of Orthopedics and Traumatology (IOT-HC-FMUSP). The FMUSP Ethics Committee has approved the study, with protocol number CAAE: 39,202,214.8.0000.0065. The study was designed and reported in accordance with the Consolidated Standards of Reporting Trials (CONSORT) guidelines.

### Participants and recruitment

The study included 43 individuals with an average age of 69.8 years (ranging from 65 to 79 years) and an average age since T2DM diagnosis of 13.6-years. The research involved the recruitment of participants from older adults community groups who met the following inclusion criteria: diagnosed with T2DM for more than a year, have been taking a stable medication (insulin or oral antidiabetics) for more than a year, glycated Hemoglobin (HbA1c) between 6.0 % and 9.0 %, no history of pain, disabling diseases, or previous surgeries that could interfere with physical training, and they must not have untreated or poorly managed chronic non-communicable diseases. The inability to participate in assessments and reevaluations during the training period and more than three absences were regarded as exclusion criteria.

All participants signed the Informed Consent Form (ICF), and then the following assessments were conducted: They first had to answer questions about age, sex, marital status, education, ethnicity, and the length of time they had a T2DM diagnosis on a sociodemographic questionnaire. After that, participants responded to the following instruments:

### Assessments

#### Laboratory analysis

At the beginning and end of treatment, peripheral blood samples (20 mL) were drawn into tubes containing EDTA anticoagulants. The plasma obtained from centrifuging whole blood with EDTA was used to measure the concentrations of fructosamine, glucose, insulin, and HbA1c.

#### Questionnaire WHOQOL-Bref

The WHOQOL-BREF questionnaire evaluates four domains: Physical, Psychological, Social Relationships, and Environment. It consists of 26 questions designed for Likert-type responses, covering intensity (“not at all” to “extremely”), capacity (“nothing” to “completely”), frequency (“never” to “always”), and evaluation (“very dissatisfied” to “very satisfied”; “very poor” to “very good”). As recommended by the WHOQOL handbook, scores for each domain were converted to a scale of 0 to 100 and expressed as percentages. Better perceived QoL is indicated by higher scores.^11^

#### Questionnaire WHOQOL-Old

The WHOQOL-Old questionnaire comprises 24 questions, categorized into six domains: sensory functioning, autonomy, past, present, and future activities, social participation, death and dying, and intimacy. Responses to the WHOQOL questions are rated on a Likert-type scale. The questions are answered using four types of scales, depending on the question's content: intensity, capacity, frequency, and evaluation.^12^

#### Montreal cognitive assessment (MoCA)

Global cognition, which includes visuospatial abilities, executive function, language, memory, attention and orientation, calculation, and abstraction, was evaluated using the MoCA instrument. Scores range from 0 to 30, with a score of less than 26 indicating cognitive impairment; the average time spent on all instruments was about forty minutes.^13^

#### Cardiopulmonary exercise testing (ergospirometry)

The maximum cardiopulmonary and metabolic functional capacity of the participants was assessed using a progressive exercise test to exhaustion, employing a ramp-style protocol on a treadmill. Targeting a total exercise duration of 8 to 15 min, each participant's physical capacity was taken into account when determining the fixed and self-selected speed, which was increased every minute. Oxygen consumption (VO_2_) and carbon dioxide production (VCO_2_) were measured via gas exchange, breath by breath, using a computerized system. During the test, systolic and diastolic blood pressure were measured every two minutes, and Heart Rate (HR) was tracked using an electrocardiogram. Blood pressure was taken at minutes 1, 2, 4, and 6 of recovery, and HR monitoring persisted until the sixth minute. Evaluations were conducted on a number of parameters, including end-tidal pressures of carbon dioxide and oxygen, respiratory rate, tidal volume, oxygen consumption, carbon dioxide production, and respiratory quotient. The lowest value of the oxygen ventilatory equivalent and points of non-linear changes in the measured variables were among the criteria used to determine the ventilatory threshold (VT1 and VT2) and maximum oxygen uptake (VO_2_ max). The authors tracked participants' subjective fatigue using the Borg scale.

### Losses to follow-up

**Fig. 1:**
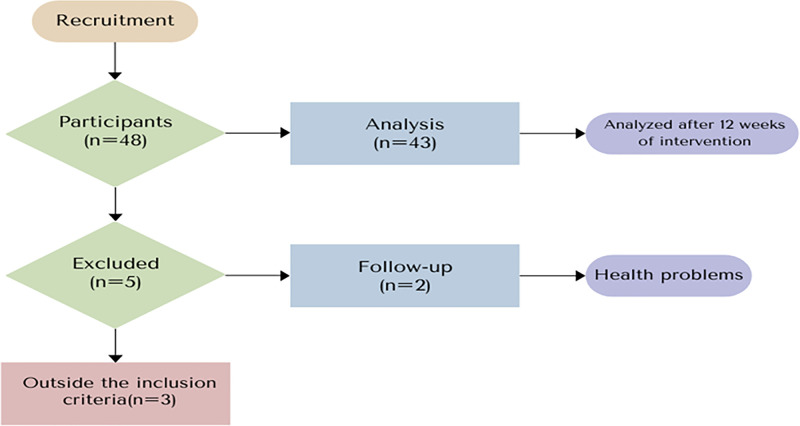
Flowchart of the study stages.

A total of 49 participants were recruited for the study. Of these, 2 were excluded for not starting the intervention. During the intervention period, 1 participant withdrew due to health reasons, and 3 were excluded for having more than three absences in the training program. At the end of the 12-week combined training intervention, 43 (91.5 %) participants completed the training (Fig. 1).

### Interventions

#### Resistance exercise training

Although there are still gaps in the literature regarding the optimal exercise order specifically for older adults with T2DM, the authors chose resistance training followed by aerobic training, as this sequence allows for better strength performance (since participants are not fatigued by prior aerobic activity).

Over a period of 12-weeks, participants engaged in two training sessions per week. The workout routine consisted of six exercises targeting major muscle groups: crunches, calf raises, leg press, leg extension, and rowing. Each exercise involved three sets of eight to twelve repetitions. The intensity of the workouts was maintained between levels 7 and 8, monitored using the Rating of Perceived Exertion (RPE) through the OMNI-Resistance Exercise Scale (0–10), a validated subjective tool for strength training.^14^ This self-reported measure was complemented by objective load adjustments, professional supervision, and the progressive approach recommended by Lagally and Robertson (2006), with training loads individually tailored according to each participant’s performance. When participants were able to complete more than 12 repetitions consistently across two sessions, the load was increased, following the guidelines established by the American College of Sports Medicine (ACSM) in 2009.

All sessions were supervised by experienced exercise professionals, who recorded attendance and monitored proper execution, ensuring strict adherence and safety. Rest periods of one to two minutes were observed between sets and exercises.

#### Interval training

The training began with a 30-minute interval aerobic workout on the treadmill, consisting of three minutes at the same speed as a low-intensity stimulus corresponding to the Cardiorespiratory Optimum Point (POC) (approximately 50 % of HRmax), followed by two minutes at the workload corresponding to LV2 as a ramp stimulus (approximately 70 % of HRmax).

This protocol was based on intensities individually determined by cardiopulmonary exercise testing. During aerobic training, a Polar F1™ heart rate monitor was used to control exercise intensity. The Borg scale was used to assess perceived exertion.

Although the total duration was 30-minutes, the interval structure with alternating intensities was chosen for its feasibility, safety, and metabolic efficacy in older adults with T2DM, as supported by recent literature.

#### Interval training

The training began with a 30-minute interval aerobic workout on the treadmill, consisting of three minutes at the same speed as a low-intensity stimulus corresponding to the Cardiorespiratory Optimum Point (POC) (approximately 50 % of HRmax), followed by two minutes at the workload corresponding to LV2 as a ramp stimulus (approximately 70 % of HRmax).

This protocol was based on intensities individually determined by cardiopulmonary exercise testing. During aerobic training, a Polar F1™ heart rate monitor was used to control exercise intensity. The Borg scale was used to assess perceived exertion.

Although the total duration was 30-minutes, the interval structure with alternating intensities was chosen for its feasibility, safety, and metabolic efficacy in older adults with T2DM, as supported by recent literature.

### Statistical analysis

The data were stored and analyzed with the JAMOVI® software. The sample was descriptively analyzed using mean, standard deviation, and median values and 95 % Confidence Intervals. Qualitative variables were presented as absolute and relative values. To determine the relationship between qualitative variables, the Chi-Square test and/or Fisher's exact test were used. The Shapiro-Wilk and Levene tests were used to assess normality, variance homogeneity, and adherence to the Gaussian distribution. For the analysis of pre- and post-intervention measures, a General Linear Model (GLM) was used. To control for the risk of Type I error due to multiple comparisons across different outcomes, the Bonferroni correction was applied to post hoc analyses.

Missing data were handled using multiple imputation methods, and all analyses were conducted following the intention-to-treat principle, including all participants initially enrolled, regardless of adherence or study completion. In addition to a significance level set at 5 % (p < 0.05), effect sizes for the analyses were calculated using partial eta squared (η² partial). Effect sizes were interpreted according to conventional thresholds: small (η² ≈ 0.01), medium (η² ≈ 0.06), and large (η² ≥ 0.14), providing insight into the clinical relevance of observed changes.

## Results

The socio-demographic data in Table 1 shows a majority of male participants (48.8 % with higher education), 67.4 % married, and 79 % self-identified as white.

**Table 1:** Characterization of study participants by sex, education level, marital status, ethnicity, age, and duration of Type 2 Diabetes Mellitus diagnosis.

		N	%	χ^2^	p-value
a**Gender**	Masculine	31	72.1	8.40	c0.004
	Feminine	12	27.9		
a**Education**	Illiterate	1	2.3	4	cp<0.001
	Complete Elementary	4	9.3		
	Complete Medium	14	32.6		
	Incomplete Higher	3	6.9		
	Graduated	21	48.9		
a**Marital status**	Single	3	6.9	3	c*p*<0.001
	Married	29	67.5		
	Divorced	6	13.9		
	Widower	5	11.7		
a**Ethnicity**	White	34	79.0	3	c*p*<0.001
	Brown	4	9.4		
	Black	3	6.9		
	Yellow	2	4.7		
	**M**		**DP**		
b**Age (years)**	69.8		4.92		
b**Diagnosis Time (years)**	13.6		7.62		

a Test Qui-Quadrado.

b Test *t* pareado.

c p < 0.05.

For the laboratory analysis results, the glycemic profile of the participants showed a statistically significant improvement in the fructosamine test (p = 0.018) with a medium effect size (partial η² = 0.07) (Table 2).

**Table 2:** Glycemic profile of older adults with type 2 diabetes mellitus.

	Reference value	Pre	Post	95 % CI	Time		η²p
		M (SD)	M (SD)		F	p	
**Fructosamine μmoL/L**	205 to 285 μmoL/L	301 (48.8)	279 (29.6)	−40.5 to −3.95	5.86	0.018^a^	0.071
**Glucose mg/dL**	70 to 99 mg/dL	141 (61.9)	133 (57.5)	−33.9 to 18.5	0.34	0.56	0.004
**HbA1c %**	> 6.5 %	7.14 (1.26)	9.87 (0.83)	−0.72 to 0.21	1.18	0.28	0.015
**Insulin μU/mL**	2.6 ‒ 24.9 µU/mL	12.9 (6.77)	11.7 (5.29)	−3.95 to 1.61	0.70	0.40	0.009

General Linear Model, ^a^ p < 0.05.

M, Mean; SD, Standard Deviation; 95 % CI, Confidence Interval; η²p, Partial eta squared.

In the WHOQOL-Bref questionnaire, there was no statistically significant improvement, however, with a small effect size Physicist, Psychological and Environment (partial η² ≈ 0.02–0.04) (Table 3).

**Table 3:** Evaluation of WHOQOL-Bref in the physical, psychological, social relationships, and environment domains of older adults with type 2 diabetes mellitus.

Domain	Pre	Post	95 % CI	Time		η²p
	M (SD)	M (SD)		F	p	
**Physicist**	68.6 (15.6)	74.0 (14.3)	−1.79 to 12.1	220	0.14	0.030
**Psychological**	67.9 (14.4)	72.5 (12.7)	−1.77 to 10.8	2.05	0.15	0.028
**Social Relationships**	61.6 (19.5)	65.0 (18.1)	−5.18 to 12.3	0.66	0.41	0.009
**Environment**	63.4 (15.3)	69.8 (15.6)	−0.74 to 13.6	3.19	0.07	0.043

General Linear Model * p < 0.05.

M, Mean; SD, Standard Deviation; 95 % CI, Confidence Interval; η²p, partial eta squared.

For the WHOQOL-Old questionnaire, there were no significant differences pre- and post-intervention (Table 4).

**Table 4:** Evaluation of WHOQOL-Old in the domains: sensory functioning; autonomy; past, present, and future activities; social participation; death and dying; and intimacy with type 2 diabetes mellitus.

	Pre	Post	95 % CI	Time		η²p
	M (SD)	M (SD)		F	p	
**Sensory Functioning**	80.8 (16.0)	82.0 (14.6)	−7.08 to 10.0	0.12	0.73	0.002
**Autonomy**	67.0 (17.9)	70.3 (15.0)	−6.29 to 12.2	0.41	0.52	0.008
**Past, Present and Future Activity**	63.3 (15.0)	65.0 (17.5)	−7.88 to 10.4	0.07	0.78	0.002
**Social Participation**	62.8 (16.5)	64.3 (14.8)	−7.00 to 10.5	0.16	0.68	0.003
**Death and Dying**	60.8 (24.6)	64.3 (24.4)	−9.07 to 18.8	0.49	0.48	0.010
**Intimacy**	61.5 (27.5)	59.8 (25.2)	−16.5 to 12.9	0.05	0.80	0.001

General Linear Model, * p < 0.05.

M, Mean; SD, Standard Deviation; 95 % CI, Confidence Interval; η²p, Partial eta squared.

Finally, in the MoCA assessment, there was a significant improvement only in the abstraction (p = 0.031) with a medium effect size (η² parcial = 0.12) and a small effect size in Delayed Recall (η² parcial = 0.04) and Orientation (η² parcial = 0.04) (Table 5).

**Table 5:** Evaluation of montreal cognitive assessment (MoCA) in older adults with type 2 diabetes mellitus.

	Pre	Post	95 % CI	Time		η²p
	M (SD)	M (SD)		F	p	
**Visuospatial/Executive**	3.8 (1.0)	4.2 (0.7)	−0.38 to 0.88	0.65	0.42	0.019
**Naming**	2.9 (0.2)	2.8 (0.3)	−0.21 to 0.26	0.04	0.83	0.00
**Attention**	5.5 (1.0)	5.6 (0.7)	−0.50 to 0.65	0.68	0.79	0.002
**Language**	2.5 (0.6)	2.8 (0.4)	−0.18 to 0.51	0.88	0.35	0.025
**Abstraction**	1.7 (0.4)	2.0 (0.0)	0.02 to 0.43	5.04	0.031^a^	0.129
**Delayed Recall**	3.5 (1.6)	4.0 (1.1)	−0.36 to 1.46	1.50	0.22	0.042
**Orientation**	5.4 (1.5)	6.0 (0.0)	−0.24 to 1.14	1.75	0.19	0.049
**Punctuation**	25.5 (3.3)	27.5 (2.1)	−3.38 to 3.67	0.00	0.93	0.000

General Linear Model, ^a^ p < 0.05.

M, Mean; SD, Standard Deviation; 95 % CI, Confidence Interval; η²p, Partial eta squared.

## Discussion

The main findings of this study indicate that, after three months of moderate CT, older adults with T2DM showed improvements in fructosamine levels, demonstrating a medium effect size (partial η² = 0.07). Additionally, small effect sizes (partial η² ≈ 0.02–0.04) were observed in QoL domains related to physical, psychological, and environmental well-being. Regarding cognitive function, a significant improvement was found in the abstraction domain (*p* = 0.031) with a medium effect size (partial η² = 0.12), while small effect sizes were noted in the delayed recall (partial η² = 0.04) and orientation (partial η² = 0.04) domains. Although these effect sizes are small, they may reflect meaningful changes in this population, especially considering the progressive nature of T2DM and aging. However, no significant effects were found in aging-specific aspects of QoL as assessed by the WHOQOL-Old questionnaire.

Following a 12-week program of moderate CT, there was a reduction in fructosamine concentrations with a medium effect size, corroborating previous findings that highlight physical exercise as an effective strategy for metabolic control in this population.^15^^,^^16^ HbA1c showed only a small effect, without statistical significance. This difference can be attributed to the nature of the biomarkers: while fructosamine reflects short-term glycemic control for two weeks, HbA1c represents a more stable average over the past three months. Furthermore, participants already had HbA1c values consistent with adequate glycemic control at the beginning of the study, which may have limited the potential to detect significant changes.^17^, ^18^ It is possible that more expressive behavioral changes, such as greater adherence to diet, exercise, and self-care, occurred only in the final stages of the intervention, enhancing the Hawthorne effect, and were not fully reflected in HbA1c levels.^19^ These findings highlight the complexity of managing T2DM and suggest that longer or more intensive interventions may be necessary to consistently modify long-term glycemic control markers.

Although no statistically significant differences were found in QoL, small effect sizes were observed in the physical, psychological, and environmental domains, indicating subtle changes. The physical domain encompasses aspects relevant to older adults, such as pain and discomfort, energy and fatigue, sleep quality and rest, mobility, daily activities, medication use, ongoing treatments, and work capacity. The presence of a small effect size may indicate that participants experienced slight improvements in physical well-being. This is consistent with literature indicating that physical exercise improves insulin sensitivity and glucose utilization,^19^ enhances muscle strength and endurance, supports bone density, and improves cardiovascular health, all factors contributing to better physical functioning and QoL.^5^^,^^20^, ^21^, ^22^, ^23^, ^24^ These benefits can facilitate daily activities, reduce fatigue, and contribute to overall well-being.

The psychological domain showed a small effect size, indicating subtle changes, second, Sousa et al.^25^ found that positive adherence to T2DM control treatment influences self-perceptions of body image, appearance, and self-esteem. Exercise causes the release of endorphins, neurotransmitters linked to mood and well-being. This endorphin release can help to alleviate anxiety, stress, and depression. Furthermore, it reduces cortisol levels, the stress hormone, promoting deeper and more restorative sleep, which is directly related to well-being.^26^ Physical exercise boosts self-esteem and confidence, and achieving goals leads to personal fulfillment.^27^

The CT was carried out in groups, providing the opportunity for social interaction, which is highly beneficial for mental health, particularly in older adults, and helps to prevent isolation and loneliness. Furthermore, it offers opportunities to learn new skills and techniques related to the chosen physical activity.^28^ Participants frequently expressed a sense of belonging, acceptance, social interaction, and access to a multidisciplinary team that included nurses, nutritionists, psychologists, physicians, physiotherapists, and physical education specialists. All were involved in disease management, offering active listening and a more humane treatment approach that focused on individuals rather than the disease. According to Pegorari et al. ,^29^ exercise can be a form of leisure, providing recreational opportunities.

The environmental domain showed a small effect size, suggesting slight improvements that may reflect meaningful perceptions among participants. This domain involves physical safety, the home environment, financial resources, the availability and quality of healthcare and social care, opportunities to learn new skills and knowledge, participation in recreational and leisure activities, the physical environment (pollution/noise/traffic/weather), and transportation. This improvement differs from the findings of Anjos et al.,^30^ possibly due to the sociodemographic characteristics of their study participants, who had lower family incomes, as opposed to the current study, which included participants with higher incomes and higher levels of education.

Although no significant improvements were observed in the WHOQOL-OLD domains, which assess aging-specific aspects such as autonomy, social participation, death and dying, and intimacy, this finding can be explained by several factors. As pointed out by Balbé et al.^31^ The natural association between aging and chronic health conditions can negatively influence these QoL dimensions. However, the participants in this study had relatively high baseline scores, limiting the possibility of detecting measurable changes. It is also important to consider that existential constructs, such as purpose in life, autonomy in the context of aging, or attitudes toward death, tend to be more stable and shaped by long-term psychosocial and cultural experiences.^32^ While physical exercise may promote indirect benefits, such as improved functionality,^20^, ^21^, ^22^, ^23^, ^24^ increased social interaction,^16^, ^28^, ^33^ and greater emotional well-being, it may not be sufficient on its own to produce substantial changes in these deeper existential domains. Such outcomes may require integrative approaches involving psychological, educational, or spiritual components.^34^ Future studies could consider combining physical training with reflective or therapeutic strategies, or even incorporating qualitative assessments, which are often more sensitive to capturing subtle but meaningful changes in the lived experience of aging.

In the study, there was a significant improvement in the abstraction domain of the MoCA, with a medium effect size, suggesting that CT may benefit executive functions, which are essential for reasoning and problem-solving and are often affected by aging and T2DM. Small effects were observed in delayed recall and orientation, while other MoCA domains showed no significant changes. These findings support the literature indicating that aerobic and resistance exercises can positively modulate aspects of executive functioning and brain plasticity in older adults, including those with T2DM.^35^ It is important to highlight that the duration of the program (12-weeks) may have limited the magnitude of the detected effects, and that cognitive functions such as memory and orientation may require longer or more specific interventions to achieve significant changes.^33^

This study presents some limitations that should be considered when interpreting the results. First, the sample was predominantly composed of male participants, with high educational attainment who self-identified as white. This homogeneity limits the generalizability of the findings to broader populations, particularly women, individuals with lower educational levels, and those from low-income backgrounds, who may respond differently to the intervention. Second, the recruitment strategy may have introduced selection bias. Despite broad dissemination through online campaigns and community-based outreach, most participants showed a pre-existing inclination toward physical activity, a common phenomenon in exercise studies. This self-selection likely resulted in a sample with higher motivation, better baseline functional capacity, and greater glycemic control, potentially reducing the magnitude of the observed effects. Moreover, individuals with more severe clinical conditions were underrepresented, further limiting the applicability of the findings to the wider population of older adults with T2DM. Finally, the absence of a control group prevents attributing the observed benefits exclusively to the CT program, as factors such as social interaction during group sessions may also have influenced the outcomes.

The finding that, despite the observed benefits, some dimensions of QOL were not improved suggests the need for a more comprehensive approach to T2DM treatment. This emphasizes the importance of conducting additional research and developing personalized strategies to address the complex interactions between health conditions and therapeutic interventions.

## Conclusion

The results indicate that a moderate-intensity CT program performed over three months led to a reduction in fructosamine levels, although no significant improvement was observed in HbA1c. Small improvements were found in QoL, measured by WHOQOL-Bref, in the physical, psychological, and environmental domains, while the aging-specific aspects assessed by WHOQOL-Old showed no significant changes. This suggests that despite the general benefits of exercise for physical and mental well-being, more specific and prolonged interventions may be necessary to deeply impact the existential and psychosocial concerns particular to older adults with T2DM.

Regarding cognitive function, the program promoted a significant improvement in abstraction performance, an important executive component, along with small effects on delayed recall and orientation. These findings indicate that the training may support neuroplasticity and the maintenance of essential cognitive functions in older adults with T2DM.

These findings highlight the complexity of diabetes management in this population and underscore the need for integrated and personalized programs that address not only metabolic aspects but also psychosocial and cognitive factors to improve QoL and overall health in older adults. Thus, the importance of multidisciplinary strategies that consider both the promotion of general well-being and the specific needs related to aging and chronic disease is reinforced, aiming for sustained improvements in QoL.

## Data availability statement

The datasets generated and/or analyzed during the current study are available from the corresponding author upon reasonable request.

## CRediT authorship contribution statement

**Matheus Henrique dos Santos Lino:** Conceptualization, Methodology, Software, Data curation, Writing – original draft, Visualization, Investigation. **Alisson de Lima Guiotto:** Data curation, Writing – original draft, Visualization, Investigation. **Vanderlei Carneiro da Silva:** Data curation, Writing – original draft, Visualization, Investigation. **Marcus Vinicius Grecco:** Conceptualization, Methodology, Software. **André Luiz de Seixas Soares:** Data curation, Writing – original draft, Visualization, Investigation. **Catherine L. Davis:** Data curation, Writing – original draft, Visualization, Investigation. **José Maria Soares-Junior:** Supervision. **Edmund Chada Baracat:** Supervision. **Guilherme Carlos Brech:** Data curation, Writing – original draft, Visualization, Investigation. **Adriana Machado Saldiba de Lima:** Conceptualization, Methodology, Software. **Júlia Maria D’Andréa Greve:** Conceptualization, Methodology, Software. **Angelica Castilho Alonso:** Conceptualization, Methodology, Software, Data curation, Writing – original draft, Visualization, Investigation, Writing – review & editing.

## Declaration of competing interest

The authors declare no conflicts of interest.
